# Serum Level of Fibroblast Growth Factor 21 Is Independently Associated with Acute Myocardial Infarction

**DOI:** 10.1371/journal.pone.0129791

**Published:** 2015-06-19

**Authors:** Wenduo Zhang, Songyun Chu, Wenhui Ding, Fang Wang

**Affiliations:** 1 Department of Cardiology, Beijing Hospital, China Ministry of Health, Beijing, P.R.China; 2 Department of Cardiology, Peking University First Hospital, Beijing, P.R.China; University of Hong Kong, CHINA

## Abstract

**Background:**

Fibroblast growth factor 21 (FGF21) has been described as a metabolic hormone critical for glucose and lipid metabolism. Previously, high levels of FGF21 were observed in patients with coronary heart disease and non-acute myocardial infarction (non-AMI). In this study, we investigated the changes in FGF21 levels in Chinese patients with AMI.

**Methodology/Principal Findings:**

We used ELISA to measure circulating FGF21 levels in 55 AMI patients and 45 non-AMI control patients on the 1^st^ day after syndrome onset. All patients were followed-up within 30 days. FGF21 levels in AMI patients were significantly higher than those in non-AMI controls (0.25 (0.16–0.34) vs. 0.14 (0.11–0.20) ng/mL, *P* < 0.001). FGF21 levels reached the maximum within approximately 24 h after the onset of AMI and remained at high for 7 days, and the FGF21 level (OR: 16.93; 95% confidence interval (CI): 2.65–108.05; *P* = 0.003) was identified as an independent factor associated with the presence of AMI. On the 7^th^ day, FGF21 levels were significantly higher in the patients who subsequently developed re-infarction within 30 days than in the patients who did not develop re-infarction (with vs. without re-infarction: 0.45 (0.22–0.64) vs. 0.21 (0.15–0.29) ng/mL, *P* = 0.014).

**Conclusions/Significance:**

The level of serum FGF21 is independently associated with the presence of AMI in Chinese patients. High FGF21 levels might be related to the incidence of re-infarction within 30 days after onset.

## Introduction

Fibroblast growth factor (FGF) 21 is a circulating hormone-like molecule secreted primarily by the liver that has been demonstrated to be a key metabolic regulator of glucolipid metabolism and insulin sensitivity [1,2]. Human studies have indicated that serum levels of FGF21 were increased in obese people and in people with metabolic syndrome or diabetes mellitus [3,4]. Recently, numerous studies have focused on the association between FGF21 and cardiovascular disease, and circulating FGF21 levels have been demonstrated to be elevated in patients with certain chronic ailments such as carotid atherosclerosis [5], coronary artery disease (CAD) [6], and hypertension [7]. In the patients with type 2 diabetes, elevated FGF21 levels were shown to be associated with increased risk of cardiovascular events over 5 years [8] and to also be predictive of combined cardiovascular morbidity and mortality during a 2-year follow-up [9]. Furthermore, in an ischemia animal model, FGF21 was found to exert a cardioprotective effect, which was diminished in obesity [10].

The aforementioned studies have indicated that FGF21 might function as a critical metabolic hormone in the cardiovascular system. Thus, investigating how FGF21 levels change in acute ischemic cardiovascular disease is of considerable interest. To examine the characteristics of FGF 21 in patients with acute myocardial infarction (AMI), we measured the dynamic change in circulating FGF21 levels in 100 Chinese patients (55 AMI patients and 45 non-AMI patients) and analyzed the association of FGF21 levels with a cluster of metabolic parameters and clinical end points.

## Study Participants and Methods

### Participants

We enrolled 55 patients in whom AMI (including ST-segment and non-ST-segment elevation, within 24 h after admission) was diagnosed between March and August 2013 at the Ministry of Health, Beijing Hospital. The control group included 45 patients with chest pain without creatine kinase (CK), creatine kinase-MB (CK-MB), and Troponin T (TNT) elevation. Coronary heart disease was also diagnosed in the patients in the control group by means of coronary angiography.

The included patients were Chinese patients aged over 18 years old in whom AMI was diagnosed according to published criteria [11]. The exclusion criteria were (1) allergy to or inability to tolerate statins; (2) stroke or a history of visceral bleeding disorders in the previous 6 months; (3) severe kidney disease and/or coagulation abnormalities; (4) a history of valvular heart disease, cardiomyopathy, myocarditis, congenital heart disease, peripheral vascular disease, or infective endocarditis, or of a combination of these ailments; (5) Stage 3–5 chronic kidney disease (CKD); (6) cancer or life expectancy of no more than 1 year; and (7) chronic heart failure and other diseases that adversely affect short-term prognosis.

The AMI patients received medication according to published guidelines [12,13]. All participants provided written informed consent, and the study was approved by the Ethics Committee of Beijing Hospital and complied with the Declaration of Helsinki.

### Anthropometric and biochemical measurements

Heart rate was measured at the time at which the patients were admitted to the hospital. The body mass index (BMI) is defined as their body mass divided by the square of their height—with the value universally being given in units of kg/m^2^. Blood pressure (BP) was measured at patient’s admission to the ward by using a mercury sphygmomanometer. Diabetes, hypertension, and dyslipidemia were diagnosed according to published guidelines [14,15,16]. Blood samples were collected after overnight fasting. Serum levels of fasting blood glucose, B-type natriuretic peptide (BNP), C-reactive protein (CRP), CK, CK-MB, TNT total cholesterol (TC), high-density lipoprotein cholesterol (HDL-c), low-density lipoprotein cholesterol (LDL-c), and triglycerides (TG) were measured using standard laboratory methods at a certified clinical examination laboratory. Blood samples were collected after overnight fasting. Fasting plasma glucose (FPG; measured after a 10-h overnight fast) was assessed using the glucose oxidase method. TC and TG levels were determined using the CHOP-PAP and GPO-PAP methods (Prodia-Diagnostics, Beijing). The concentration of LDL-c was analyzed using the selective solubilization method (LDL-c test kit, Kyowa Medex, Tokyo), and the concentration of HDL-c was determined using a homogeneous method (Determiner L HDL, Kyowa Medex). CK and CK-MB activity were measured in venous blood samples within 24 h after onset by using an immunoinhibition assay (CK and CK-MB kit, Ausbio, Beijing), and the level of BNP was determined using an electrochemiluminescence immunoassay (Roche). cTNT was measured using a commercial kit (Roche Diagnostics, Rotkreuz, Switzerland). Serum CRP was measured using the particle-enhanced immunonephelometry assay (Dade Behring Inc., Newark, NJ, USA).

Serum FGF21 levels on the 1^st^, 3^rd^, and 7^th^ day from syndrome onset were measured using commercial ELISA kits, as per manufacturer instructions (Antibody and Immunoassay Services, University of Hong Kong).

### Endpoint parameters

The clinical endpoints were all-cause death, myocardial re-infarction, and all-cause readmission within 30 days.

### Statistical analysis

All statistical analyses were performed using Statistical Package for Social Science version 19.0 (SPSS, Inc., Chicago, IL). Normally distributed data were expressed as means ± SD, and skewed data were expressed as medians with the interquartile range. Intergroup comparisons of clinical values were performed using unpaired Student’s *t* test, and skewed data were log-transformed before analyses. The chi-square test was performed for intergroup comparisons of categorical variables. Partial correlation analysis was used to examine the correlation between FGF21 levels and clinical parameters, and multivariate logistic regression analysis was used to examine the independent factors of CAD occurrence. The variables input into the multiple logistic regression were variables that differed significantly between patients with and without AMI, and the diagnostic parameters were not included. All *P* values were two-tailed and *P* < 0.05 was considered statistically significant.

## Results

### Serum FGF21 levels and clinical parameters at baseline

Table 1 presents the characteristics of AMI patients (n = 55) and control patients (n = 45). The age of the patients with and without AMI was comparable (64 ± 11 vs. 63 ± 10 y, *P* = 0.720), but the AMI group contained more males than did the non-AMI group (51% vs. 21%, *P* = 0.005). Although the BMI in AMI patients was slightly lower than that in control patients, the difference was not significant after adjusting for sex (*P* = 0.285). As expected, compared to control patients, AMI patients had higher fasting glucose levels (8.9 ± 2.6 vs. 6.0 ± 2.3 mmol/L, *P* < 0.001) and a higher rate of smoking (66.7% vs. 31.2%, *P* < 0.001). The lipid profile of the patients in the 2 groups was similar. Serum FGF21 levels on admission were significantly higher in AMI patients than in control patients (0.25 (0.16–0.34) vs. 0.14 (0.11–0.20) ng/mL, *P* < 0.001).

**Table 1:** Baseline clinical parameters of patients, sorted according to the presence or absence of AMI.

Variable	Control (n = 45)	AMI (n = 55)	*P*
**Age, y**	63 ± 10	64 ± 11	0.720
**Male, n (%)**	27 (56.3)	51 (81.0)	0.005
**BMI (kg/m** ^**2**^ **)**	27.4 ± 6.1	25.2 ± 2.5	0.035
**Fasting glucose (mmol/L)**	6.0 ± 2.3	8.9 ± 2.6	<0.001
**Diabetes, n (%)**	16 (33.3)	22 (34.9)	1.000
**Hypertension, n (%)**	30 (62.5)	41 (65.1)	1.000
**Smoking, n (%)**	15 (31.2)	42 (66.7)	<0.001
**Hyperlipidemia, n (%)**	30 (62.5)	40 (63.5)	0.835
**Stroke, n (%)**	4 (8.3)	7 (11.1)	1.000
**Heart Rate (bpm)**	68.7 ± 10.9	76.4 ± 16.2	0.006
**TC (mmol/L)**	4.1 ± 1.3	4.4 ± 1.3	0.218
**LDL-c (mmol/L)**	2.5 ± 1.0	2.8 ± 1.0	0.106
**HDL-c (mmol/L)**	1.0 ± 0.2	1.0 ± 0.2	0.424
**TG (mmol/L)**	1.5 ± 0.9	1.7 ± 1.3	0.364
**FGF21 (ng/ml)** *	0.14 (0.11–0.20)	0.25 (0.16–0.34)	<0.001

AMI: acute myocardial infarction

Values are expressed as mean ± standard deviation, median with interquartile range, or n (%)

*Log-transformed before analysis

**Fig 1:**
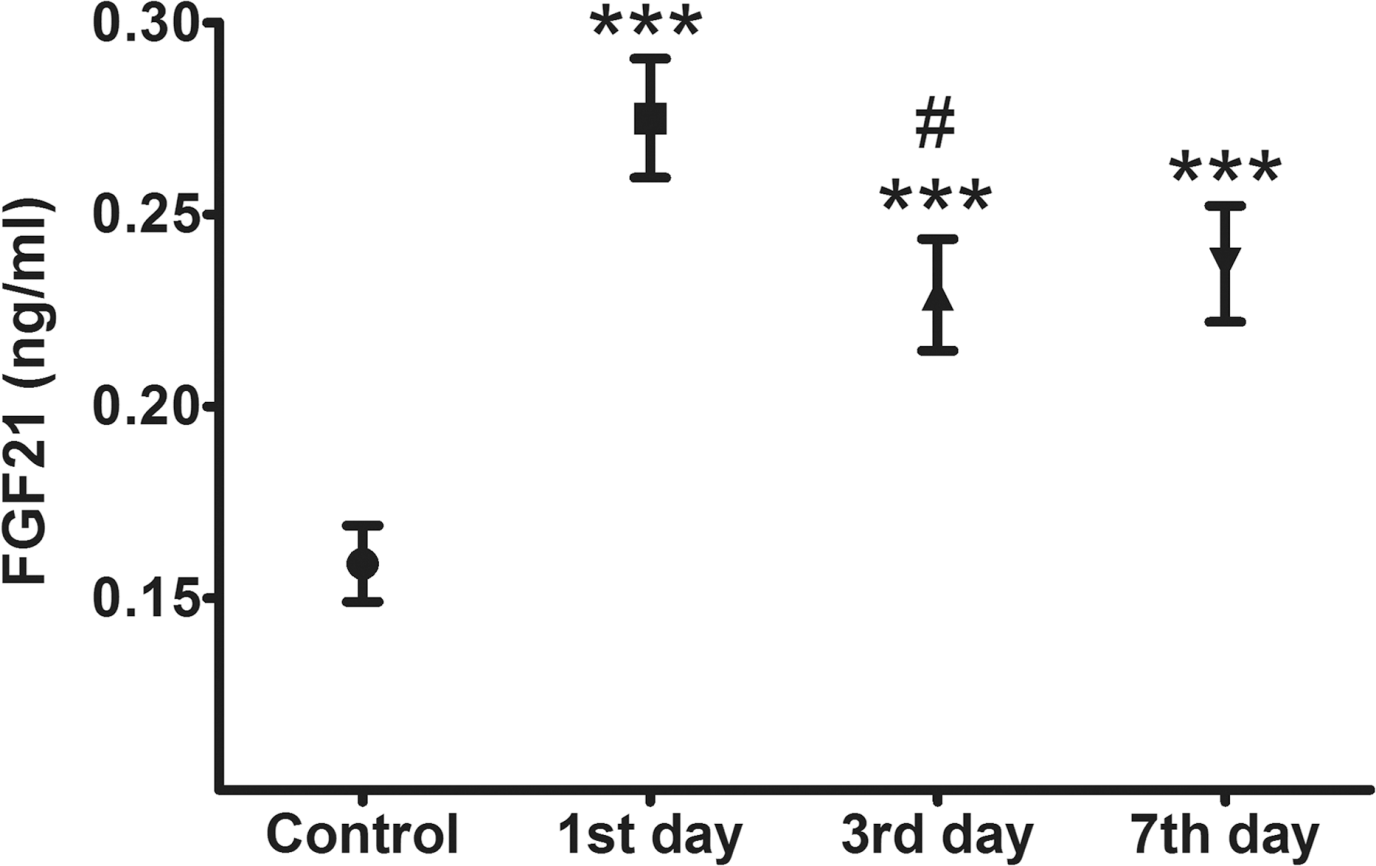
Serum FGF21 levels in patients with acute myocardial infarction (n = 55) on the 1^st^, 3^rd^, and 7^th^ day after admission, compared with control patients (n = 45). Values are shown as medians with the interquartile range. Data were log-transformed before analysis. ***P < 0.001 compared with controls; ^**#**^ *P* < 0.05, compared with FGF21 levels on the 1^**st**^ day after admission.

The high serum FGF21 levels in AMI patients on admission were maintained for 1 week (Fig 1). On the 1^st^ day after AMI diagnosis, the FGF21 levels were 0.25 (0.17–0.34) ng/mL, and on the 3^rd^ day, the levels were decreased slightly (0.20 (0.15–0.29) ng/mL, *P* = 0.032). However, from the 3^rd^ to the 7^th^ day, the levels of FGF21 showed almost no change (0.21 (0.15–0.29) ng/mL, *P* = 0.701). The FGF21 levels measured at these 3 time points were closely correlated (all *P* < 0.05).

Univariate analysis (Table 2) revealed significant correlations between the levels of FGF21 and HDL-c (*P* = 0.002) and BNP (*P* = 0.009) in AMI patients, and, as expected, the FGF21 level was closely correlated with the levels of fasting glucose (*P* = 0.056) and TG (*P* = 0.007) in the non-AMI group. However, FGF21 levels were not correlated with the levels of CK, CK-MB, and TNT. Interestingly, FGF21 levels appeared to be positively correlated with heart rate in both patient groups (*P* = 0.073 in AMI patients, *P* = 0.060 in controls).

**Table 2:** Correlation of FGF21* with baseline parameters in AMI and control patients.

	AMI (n = 55)		Control (n = 45)	
	β	*P*	β	*P*
**Age**		0.936		0.267
**Sex**		0.398		0.112
**BMI**		0.212		0.678
**Fasting glucose**		0.824	0.290	0.056
**TC**		0.520		0.796
**LDL-c**		0.462		0.428
**HDL-c**	-0.424	0.002		0.870
**TG** *		0.200	0.423	0.007
**HR**	0.243	0.073	0.286	0.060
**CK** *		0.242		
**CK-MB** *		0.532		
**BNP** *	0.398	0.009		
**TNT** *		0.147		
**CRP** *		0.572		

β: standardized regression coefficient

*Log-transformed before analysis

### Serum FGF21 was independently associated with AMI at baseline

Multiple logistic regression analysis (Table 3) showed that FGF21 was independently associated with the presence of AMI at baseline (OR: 16.93; 95% confidence interval (CI): 2.65–108.05; *P* = 0.003), as were fasting glucose (OR: 1.43; 95% CI: 1.06–1.95; *P* = 0.021) and current smoking (OR: 5.40; 95% CI: 1.27–23.03; *P* = 0.023). The OR values suggest that the independent association of FGF21 with AMI is stronger than the associations of the traditional risk factors smoking and fasting glucose levels.

**Table 3:** Multiple logistic regression analysis showing factors independently associated with AMI.

Parameters	OR	95% CI	*P*
Fasting glucose	1.43	1.06–1.95	0.021
Smoking	5.40	1.27–23.03	0.023
FGF21*	16.93	2.65–108.05	0.003

Variables included in the original model (backward stepwise) were age, sex, BMI, fasting glucose, LDL-c, smoking, and FGF21 expression level

*Log-transformed before analysis

### Serum FGF21 levels might be related to short-term prognosis

Among the 55 AMI patients, 4 died, 5 were re-hospitalized, and 3 were re-infracted within 30 days (Table 4). A comparison of the FGF21 levels between the patients who experienced or did not experience adverse events suggested that the FGF21 level might be a predictor for such events.

**Table 4:** Comparison of clinical parameters between patients with death, re-admission, and re-infarction (P values).

	Death	Re-admission	Re-infarction
With/without	4/51	6/49	3/52
**Age**	0.178	0.785	0.225
**Sex (Male)**	0.573	0.829	0.367
**BMI**	0.106	**0.034**	0.319
**Fasting glucose**	0.130	0.991	0.155
**smoking**	0.209	0.935	0.497
**LDL-c**	0.328	0.604	**0.081**
**HDL-c**	0.575	**0.048**	0.417
**TG** *	0.263	0.722	0.289
**CK** *	0.648	0.485	0.707
**CK-MB** *	0.728	0.367	0.798
**BNP** *	**0.096**	0.590	**0.009**
**TNT** *	0.885	0.468	0.551
**CRP** *	0.366	0.269	0.146
**FGF21 1** ^**st**^ **day** *	0.259	**0.096**	**0.053**
**FGF21 3** ^**rd**^ **day** *	0.970	0.707	0.731
**FGF21 7** ^**th**^ **day** *	**0.061**	0.466	**0.014**

*Log-transformed before analysis

As shown in S1 Table, FGF21 levels on the 1^st^ and 7^th^ day were significantly higher in the patients who subsequently developed re-infarction during 30 days than in those who did not (1^st^ day: 0.45 (0.28–0.51) vs. 0.24 (0.17–0.32), *P* = 0.056; 7^th^ day: 0.45 (0.22–0.64) vs. 0.21 (0.15–0.29), *P* = 0.014), which was similar to the case of the recognized risk factor BNP (9252.0 (7614.0–25710.0) vs.1054.0 (356.0–2792.0), *P* = 0.009) (Table 4). In the patients who died, FGF21 levels, like BNP levels, on the 7^th^ day after admission were also higher than those in the survivors, although both values did not reach statistical significance (FGF21: 0.34 (0.18–0.59) vs. 0.21(0.15–0.29), *P* = 0.061; BNP: 8433.0 (2031–2159.0) vs.1054.0 (355.9–2792.0), *P* = 0.096).

## Discussion

In this study, we first demonstrated that FGF21 levels in AMI patients were markedly elevated when compared to the level in non-AMI patients. The following findings suggested that the FGF21 expression level was correlated with AMI.

First, we confirmed that circulating FGF21 was increased in a statistically significant manner in patients with CAD in the acute status, in addition being elevated in patients with chronic CAD as shown previously [17]. Furthermore, we demonstrated that the serum FGF21 level was considerably higher on the 1^st^ day after onset in AMI patients than in non-AMI CAD patients and that it remained high for 7 days, although the level was slightly lower by the 7^th^ day when compared with that at the beginning.

Second, we found that in AMI patients, FGF21 levels were closely correlated with those of HDL-c and BNP but not LDL-c, fasting glucose, and TG, which was distinct from the results obtained for non-AMI CAD patients and from the results of previous studies [17]. Serum BNP level is recognized to be elevated in AMI patients and to be closely related with acute and chronic infarct size and myocardial function after AMI [18,19], and BNP is considered to function as a protective factor for coronary plaque components, chronic infarct size, and myocardial function after AMI [20,21]. As in the case of BNP, mRNA expression levels of FGF21 were demonstrated to be increased in rat cardiac micro-vascular endothelial cells cultured under atherosclerosis-like conditions [5,22], and the addition of exogenous FGF21 potently inhibited the apoptosis of cardiac endothelial cells [22]. In a rat model, cardiac FGF21 was expressed and secreted in an autocrine-paracrine manner in response to obesity and hypoxia, and ischemia upregulated this FGF21 expression and secretion [10]. The results of this study in humans agree with the findings of the animal study and suggest that FGF21 expression might be a crucial response that counters ischemic injury. However, the origin of the elevated levels of circulating FGF21during AMI remains unknown.

Third, FGF21 levels on the 1^st^ and 7^th^ days were higher in patients with re-infarction than in patients without re-infarction. Furthermore, the level of FGF21 on the 7^th^ day was associated with death within 1 month after AMI; this agrees with the finding of a previous study that showed that the FGF21 level can predict morbidity and mortality in coronary heart disease [9].

The aforementioned results suggest that FGF21 levels could serve as an early indicator of repeated infarction and that FGF21 upregulation might function as a compensatory response to injury induced by ischemia. A previous study showed that FGF21 was produced and secreted by cardiomyocytes [23] in response to cardiac ischemic stress, and the secreted cardiac FGF21 inhibited isoproterenol-induced cardiac hypertrophic damage. In the mouse model of myocardial ischemia/reperfusion injury, FGF21 was upregulated, and this reduced cell death and myocardial infarction in association with an improvement of myocardial function.

The mechanism of action of FGF21 involves the upregulation of glucose transporter protein-1 (GLUT-1) expression, which promotes glucose uptake and metabolism by fat cells and long-term energy storage [24]. Moreover, FGF21 inhibits hepatic glycogen degradation and thereby reduces the levels of circulating glucagon and helps maintain the tight hormonal balance required for ensuring normal physiology [25]. However, in heart tissue, the FGF21 signaling pathway might involve the activation of the PI3K/Akt (phosphatidylinositol 3-kinase/Akt), ERK1/2(extracellular signal-regulated kinase), and AMPK (AMP-activated protein kinase) pathways [10]. Furthermore, the heart also appears to be a target of locally produced FGF21, even though FGF21 is an endocrine FGF. A previous study showed that FGF21 was released from adipose tissue in myocardial injury, and this contributed to myocardial protection by acting through the FGFR1/b-Klotho–PI3K–Akt1–BAD signaling network [26]. Although locally produced FGF21 exerts cardioprotective effects, several of the steps that link elevated circulating FGF21 levels and re-infarction remain unknown.

In this study, we also determined that FGF21 levels were closely related with heart rate in both AMI and non-AMI patients; however, this might only be a trend that was observed because the sample size was small, and the underlying mechanism warrants further investigation. However, collectively, our results suggest that FGF21 can act as a critical protective factor after AMI in humans. Furthermore, the results of multiple logistic regression analysis revealed that the FGF21 level—like fasting glucose levels and current smoking—was independently associated with the presence of AMI at baseline, and the calculated OR values further suggested that the association of FGF21 with the AMI was stronger than those of smoking and fasting glucose levels.

The limitations of this study are the following. First, the sample size was small, especially in relation to death and re-infarction, and a large sample size might be required for investigating the observed effect comprehensively. Second, the study population was homogenous: the participants were middle- and old-aged Chinese adults presenting at a single health institute and a focused clinical-care department (cardiology). Third, certain inflammatory factors were not tested and the changes of CK and BNP were not recorded; obtaining these data might help identify the mechanism underlying the increase in FGF21 in AMI development and progression.

In conclusion, this study has shown that the serum FGF21 level was markedly increased on the 1^st^ day after onset in AMI patients and that the level remained high on the 3^rd^ and 7^th^ days. The levels of FGF21 were closely related with those of BNP. The high concentration of circulating FGF21 was associated with the incidence of AMI independently of the effects of age, sex, BMI, fasting glucose levels, and LDL-c. Our findings suggest that FGF21 might be involved in cardioprotective effects in AMI and might also serve as a novel biomarker for the prognosis of AMI.

## Supporting Information

S1 Table Clinical parameters listed according to follow-up status. (DOCX)
