# Supplementary material for: Serum Level of Fibroblast Growth Factor 21 Is Independently Associated with Acute Myocardial Infarction
Source: PLoS One. 2015 Jun 19;10(6):e0129791. doi: 10.1371/journal.pone.0129791 (PMC4474722; doi:10.1371/journal.pone.0129791)
Supplement: S1 Table — (DOCX) [file pone.0129791.s001.docx]

**Supporting information Table S1 Clinical parameters listed according to follow-up status**

|  | **Death** | | | **Re-admission** | | | **Re-infarction** | | |
| --- | --- | --- | --- | --- | --- | --- | --- | --- | --- |
|  | **no** | **yes** | ***P*** | **no** | **yes** | ***P*** | **no** | **yes** | ***P*** |
| **n** | **51** | **4** |  | **49** | **6** |  | **52** | **3** |  |
| **Age** | 64 ± 10 | 72 ± 11 | 0.178 | 65 ± 11 | 66 ±6 | 0.785 | 64 ± 10 | 72 ± 10 | 0.225 |
| **Sex (Male)** | 40 | 4 | 0.573 | 39 | 5 | 0.829 | 40 | 3 | 0.367 |
| **BMI** | 25.5 ± 2.5 | 23.4 ± 1.8 | 0.106 | **25.6 ± 2.5** | **23.3 ± 1.6** | **0.034** | **25.5 ± 2.5** | **24.0 ± 1.5** | 0.319 |
| **Fasting glucose** | 9.1 ± 2.6 | 6.7 ± 2.0 | 0.130 | 8.9 ± 2.3 | 8.9 ± 4.4 | 0.991 | 9.2 ± 2.5 | 6.6 ± 2.8 | 0.155 |
| **smoking** | 34 | 1 | 0.209 | 31 | 4 | 0.935 | 33 | 1 | 0.497 |
| **LDL-c** | 2.8 ± 0.9 | 2.2 ± 1.2 | 0.328 | 2.8 ± 1.0 | 2.6 ± 0.9 | 0.604 | **2.8 ± 0.9** | **1.6 ± 0.8** | **0.081** |
| **HDL-c** | 1.0 ± 0.2 | 0.9 ± 0.3 | 0.575 | **1.0 ± 0.2** | **0.8 ± 0.1** | **0.048** | 1.0 ± 0.2 | 0.9 ± 0.4 | 0.417 |
| **TG*** | 1.42  (0.93–2.28) | 0.98  (0.54–1.21) | 0.263 | 1.27  (0.90–2.41) | 1.43  (1.19–1.93) | 0.722 | 1.4  (0.9–2.3) | 0.8  (0.5–1.0) | 0.289 |
| **CK*** | 1024.0  (316.0–2849.0) | 589.5  (328.0–2232.5) | 0.648 | 941.0  (335.0–2642.5) | 3039.0  (208.8–4406.8) | 0.485 | 1024.0  (354.0–2849.0) | 590.0  (241.0–2780.0) | 0.707 |
| **BNP*** | **1054.0**  **(355.9–2792.0)** | **8433.0**  **(2031–2159.0)** | **0.096** | 1056.0  (327.2–3258.0) | 1607.5  (367.5–8023.5) | 0.590 | **1054.0**  **(356.0–2792.0)** | **9252.0**  **(7614.0–25710.0)** | **0.009** |
| **TNT*** | 19.2  (9.1–90.8) | 49.0  (3.2–94.8) | 0.885 | 17.8  (8.6–89.4) | 91.1  (3.4–94.3) | 0.468 | 19.2  (9.1–90.8) | 94.0  (4.0–95.0) | 0.551 |
| **CRP*** | 1.38  (0.35–8.32) | 6.69  (0.9–13.0) | 0.366 | 0.93  (0.36–9.1) | 5.5  (1.2–12.0) | 0.269 | 0.93  (0.35–0.99) | 11.85  (1.53–13.6) | 0.146 |
|  |  |  |  |  |  |  |  |  |  |
| **FGF21 1^st^ *** | 0.25  (0.17–0.32) | 0.37  (0.19–0.50) | 0.259 | 0.24  (0.17–0.33) | 0.31  (0.25–0.47) | **0.096** | **0.24**  **(0.17–0.32)** | **0.45**  **(0.28–0.51)** | **0.053** |
| **FGF21 3^rd^ *** | 0.21  (0.13–0.29) | 0.16  (0.15–0.41) | 0.970 | 0.20  (0.14–0.29) | 0.18  (0.14–0.29 | 0.707 | 0.20  (0.13–0.29) | 0.16  (0.15–0.49) | 0.731 |
| **FGF217^th^ *** | **0.21**  **(0.15–0.29)** | **0.34**  **(0.18–0.59)** | **0.061** | 0.21  (0.14-0.28) | 0.26  (0.15-0.35) | 0.466 | **0.21**  **(0.15–0.29)** | **0.45**  **(0.22–0.64)** | **0.014** |

Values are expressed as mean ± standard deviation or median with the interquartile range

*Log-transformed before analysis
